# A Genetic Strategy for Stochastic Gene Activation with Regulated Sparseness (STARS)

**DOI:** 10.1371/journal.pone.0004200

**Published:** 2009-01-15

**Authors:** Sheng-zhi Wang, Bao-hua Liu, Huizhong W. Tao, Kun Xia, Li I. Zhang

**Affiliations:** 1 Zilkha Neurogenetic Institute, University of Southern California, Los Angeles, California, United States of America; 2 Department of Physiology & Biophysics, Keck School of Medicine, University of Southern California, Los Angeles, California, United States of America; 3 Department of Cell and Neurobiology, Keck School of Medicine, University of Southern California, Los Angeles, California, United States of America; 4 The State Key Laboratory of Medical Genetics, Central South University, Changsha, Hunan, People's Republic of China; University of Edinburgh, United Kingdom

## Abstract

It remains a challenge to establish a straightforward genetic approach for controlling the probability of gene activation or knockout at a desired level. Here, we developed a method termed STARS: stochastic gene activation with genetically regulated sparseness. The stochastic expression was achieved by two cross-linked, mutually-exclusive Cre-mediated recombinations. The stochastic level was further controlled by regulating Cre/*lox* reaction kinetics through varying the intrachromosomal distance between the *lox* sites mediating one of the recombinations. In mammalian cell lines stably transfected with a single copy of different STARS transgenes, the activation/knockout of reporter genes was specifically controlled to occur in from 5% to 50% of the cell population. STARS can potentially provide a convenient way for genetic labeling as well as gene expression/knockout in a population of cells with a desired sparseness level.

## Introduction

To address many questions in neurobiology, developmental biology and cancer biology, it becomes more and more important to generate genetic models in which transgene (e.g. fluorescence markers) expression or inactivation of gene of interest occurs only in a small number of cells. This poses two technical challenges to molecular geneticists: first, how to manipulate gene activation in a stochastic way; second, how to control the stochastic level of gene activation. The establishment of such mechanisms will allow us to achieve differential gene expression in cells with otherwise identical “transcriptome” profiles, and also to control the percentage of cells that have specific gene expression/knockout in a block of tissue, i.e. the level of sparseness. Several genetic methods have been developed for achieving gene expression/knockout in a small population of cells: 1) screening transgenic lines with variegated gene expression [1]–[3]; 2) CreER-*lox* mediated intrachromosomal recombination [2], [4]–[6]; and 3) Cre-*lox* mediated interchromosomal recombination during mitosis [7]–[12] (e.g. Mosaic analysis with double markers in mice, MADM). The application of variegation effect depends on screening of transgenic lines with the desired expression pattern. The method of CreER will require careful titration of the dose of tamoxifen for the specific tissue under examination [5], [6]. As for MADM, the required mitotic recombination, although facilitates tracing of cell lineage, makes it less efficient to label differentiated cells. In all these methods, a straightforward genetic control of the percentage of cells with expression/knockout of gene of interest cannot be readily achieved. Recently, a Brainbow transgenic strategy has been developed [13], in which Cre/*lox* recombination was utilized to create a stochastic choice of expression among three different fluorescence proteins. Cells can be labeled with as many as tens of different colors according to the combination of differential doses of fluorescence proteins expressed in each cell. This method enables imaging a large number of individual cells in the same circuit. In the present study, by exploiting the Cre-*lox* mediated intrachromosomal recombination and implementing a novel mechanism to control the stochastic level, we developed a new strategy for purely genetic control of gene activation with desired sparseness.

## Results and Discussion

**Figure 1:**
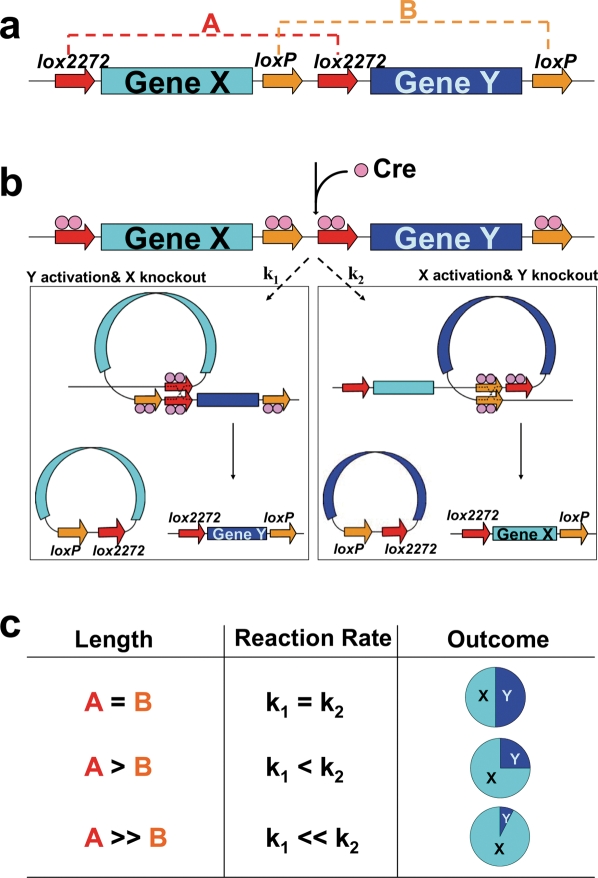
STARS strategy. a, Schematic drawing of the structure of STARS transgene: gene X flanked by *lox2272* pair (Unit A) and gene Y flanked by *loxP* pair (Unit B) are crossly linked and subjected to Cre action. b, Two Cre molecules bind to each *lox* site and two identical *lox* sites are brought together for the recombination to occur. Cre stochastically excises recombination unit A or B (the reaction kinetics reflected by the rate constant k_1_ and k_2_, respectively) and leads to mutually exclusive expression of gene Y and gene X. c, We hypothesize that the length of recombination unit may affect the reaction kinetics and leads to differential outcomes after Cre action.

To develop a method that can stochastically activate gene expression with desired sparseness, we first considered the reaction kinetics of Cre/*lox*-mediated intrachromosomal recombination. There are two ways that can potentially regulate the Cre/*lox* reaction kinetics. First, *in vitro* studies suggest that mutations in the spacer region of *loxP* result in variations of recombination efficiency [14]. However, Brainbow transgene expression in cell lines or *in vivo* did not reveal apparent differences in recombination efficiency among the three *lox* variants used [13]. Second, a previous *in vivo* study [15] demonstrated that Cre can effectively induce intrachromosomal recombination between two *loxP* sites separated as far as several mega basepairs on mouse chromosome 11 and that this efficiency is reduced when the substrate length increases. We hypothesize that as the length of DNA sequence between two *lox* sites increases, it may take a longer time for the two *lox* sites to be brought together to form the synapsed structure [16], which is required for recombination to occur. In this study, we focused on the second mechanism, and designed our strategy for stochastic gene activation/knockout with regulated sparseness (STARS) as shown in **Fig. 1a**: two independent recombination units (A and B, which contain different *lox* variants, *lox2272* and *loxP*, respectively.) are crossly linked with the first one containing gene X and the other containing gene Y. Because Cre-mediated recombination can only occur between two identical *lox* sites [13], [14], [17], [18], and excision by one recombination event removes a *lox* site necessary for the other to occur, the choice between X and Y expression is made stochastic and mutually exclusive (**Fig. 1b**). Under our hypothesis, if the lengths of the two recombination units (A and B) are equal, their reaction kinetics, reflected by the rate constant k_1_ and k_2_ respectively, should be comparable and the chances of X and Y expression should be similar. On the other hand, if the sequence in unit A is longer than that in unit B, the reaction time needed for excising A will be longer than that for B, hence the recombination will be more likely to occur in B, resulting in a higher probability of expressing gene X than Y (**Fig. 1c**). Thus, by varying the length of one unit while keeping the other constant, we can regulate the probability of the desired recombination, and control the sparseness level of gene activation.

**Figure 2:**
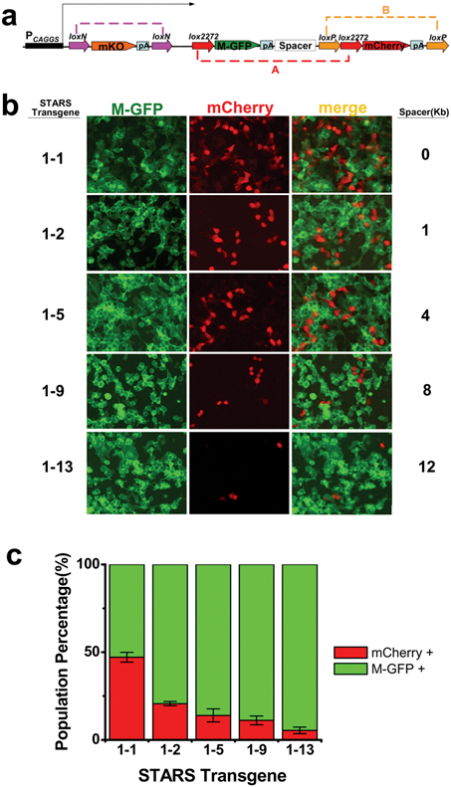
Test of STARS. a, Schematic drawing of the STARS construct for making HEK293 stable cell lines. CMV β-actin enhancer (CAGGS) promoter was used to drive the expression of STARS transgene. mKO flanked by *loxN* pair was used to mark the stable cell lines and prevent the expression of M-GFP without Cre action. The spacer following M-GFP-pA regulates the length of unit A. b, Example images of HEK293 cells stably expressing various STARS transgenes after Cre action. The names of STARS constructs and the corresponding spacer length are indicated. c, Average percentage of cells expressing mCherry (red) or M-GFP (green) in each cell population. Data from five independent experiments for each cell line were averaged. Bars represent standard deviation. Significant difference (p<0.02) was found between each pair of lines except between STARS 1–5 and 1–9 (p>0.05 for 1–5 and 1–9, p<0.02 for 1–9 and 1–13, and p<0.01 for all the remaining pairs, Tukey's multiple comparison test, n = 5).

To test this idea, a set of constructs were made, which contained membrane bound-GFP (M-GFP as gene X) flanked by *lox2272* sites, mCherry (gene Y) flanked by *loxP* sites, and a spacer in unit A of various lengths. Here, M-GFP was defined as the gene of interest and mCherry was defined as the reporter gene. We predicted that an increase in the spacer length would result in a higher probability of Cre-mediated recombination in unit B, leading to the removal of mCherry cassette. A third marker, fluorescent protein mKusabira orange(mKO) flanked by *loxN* pair (*loxN*-mKO-pA-*loxN*) was incorporated upstream of the STARS cassette to facilitate selection of cell lines that have the construct integrated in the genome, and to prevent cells from expressing M-GFP in the absence of Cre (**Fig. 2a**). Single copy of each of these constructs was inserted into the same locus of HEK293 cell genome with a targeted method (see **methods**). Selected stable cell lines for each construct were transfected with the plasmid expressing Cre recombinase and imaged five days post transfection. In the absence of Cre, all cells were mKO-positive, and no expression of M-GFP or mCherry was observed (data not shown). Five days after Cre transfection, cells expressing M-GFP and mCherry were found to be separate populations, consistent with the stochastic and mutually exclusive choice of expression and supporting the notion that the selected cell lines had only single copy STARS transgene in the genome (**Fig. 2b**). In addition, all the cells that were either M-GFP or mCherry positive lacked mKO fluorescence (data not shown), suggesting that 5 days is sufficient for the turnover of mKO protein.

**Figure 3:**
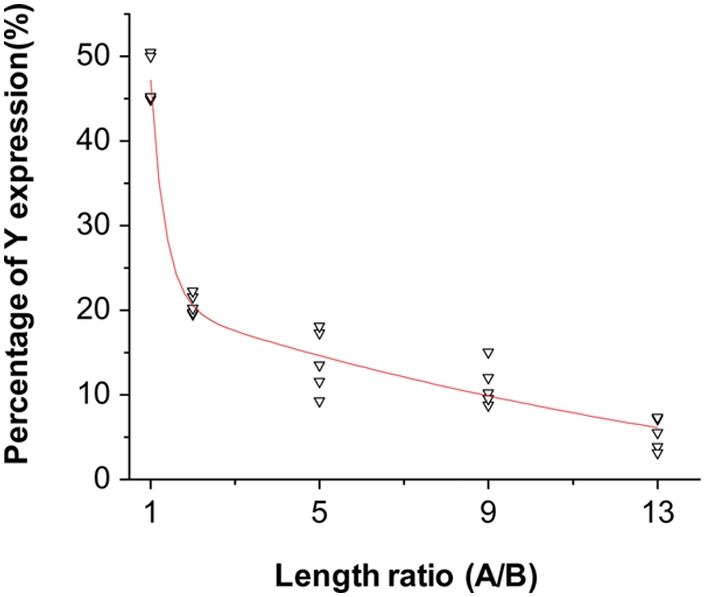
Relationship between the level of sparseness and the ratio of length between recombination units. In our STARS application, the gene Y in unit B was used as a reporter with a fixed length of about 1 kb. The regulation is achieved through unit A. Here, X-axis represents the ratio of DNA length between unit A and B. Y-axis represents the percentage of cells expressing gene Y (mCherry). Data point represents each individual experiment. The red curve represents the best fit (*r* ^2^ = 0.97) with a second order exponential function: y = y_0_+a_1_* e∧(−x/t_1_)+a_2_ * e∧(−x/t_2_), where y_0_ is 0, a_1_ is 556.79, t_1_ is 0.32, a_2_ is 23.81, t_2_ is 10.00.

To examine the effect of intrachromosomal length on the recombination efficiency, we calculated the ratio between the number of cells expressing M-GFP and mCherry. For the cell line containing the transgene without the spacer (STARS1-1), M-GFP and mCherry were expressed in roughly equal number of cells after Cre transfection (53%±3% vs. 47%±3%, respectively, mean±SD) (**Fig. 2b & 2c**), consistent with the results of Brainbow transgene expression [13]. Interestingly, as the length of the spacer in unit A increased from 0 kb to 12 kb (STARS 1-1 to STAR1-13), the number of mCherry-positive cells decreased monotonically from 47% to 5% of the total cell population, while M-GFP positive cells increased to 95% (**Fig. 2b & 2c**). Here, each cell line with a specific STARS transgene was tested five times independently. Our data indicated that, by varying the length of DNA fragment flanked by *lox2272* sites up to 13 kb, mCherry activation (or M-GFP knockout in another word) could be controlled to occur in from around 50% to 5% of cells (**Fig. 2c**). Because all STARS transgenes were integrated into the same locus of HEK293 cell genome, the differential sparseness is unlikely attributed to variations in Cre efficiency. These results support our hypothesis that the longer DNA fragment flanked by *lox* pair, the longer time it takes for Cre-mediated recombination to occur. The relationship between the percentage of cells expressing mCherry and the ratio between the lengths of two recombination units can be best described with a second order exponential decay curve (see **Fig. 3**). This curve may provide guidance for construct design to achieve desired sparseness level.

**Figure 4:**
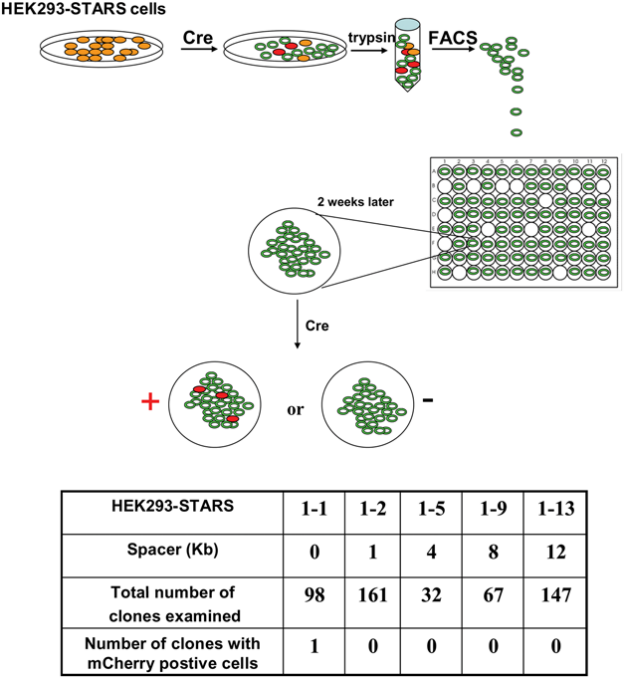
Examination of the identities of M-GFP positive cells. After Cre action on HEK293-STARS cell lines, M-GFP positive cells were sorted out and seeded at single cell/well in 96-well plates for proliferation. When the colonies contain thousands of cells (takes about 2 weeks), they were transfected with the plasmid expressing Cre. Clones that have cells expressing mCherry in 5 days post transfection were counted as positive and summarized in the table.

Because Cre expression is not long-lasting in transfected cells, possibility exists that some M-GFP positive cells resulted from simple excision of the mKO unit without recombination in the mCherry unit. This can lead to an overestimation of the number of M-GFP expressing cells that directly result from the stochastic choice between competing M-GFP and mCherry units. To estimate the number of M-GFP cells with mKO excision only (i.e. “false” M-GFP cells), we sorted out M-GFP-positive cells after Cre action by using FACS and seeded them in 96-well plates at single-cell per well (see **methods**). After cells had proliferated and expanded to colonies of thousands of cells, we further transfected individual colonies with Cre. As “false” M-GFP cells have an intact STARS cassette, Cre expression will lead to the expression of mCherry in at least some cells of the colony. This is due to a *bona fide* action of Cre on the STARS cassette. As shown in **Fig. 4**, from all the colonies collected, only one colony exhibited mCherry expressing cells. This result indicated that Cre action was in fact highly efficient and quite complete, and that the overestimation of M-GFP expressing cells was negligible.

STARS strategy can allow three types of applications. First, by combining tissue or cell-type specific Cre expression, a genetic “Golgi”-like staining of a small number of cells of a particular type can be achieved. Second, overexpression of exogenous genes in a small population of cells (e.g. by replacing mCherry with any gene of interest) can be achieved. Finally, it can provide a potential way for sparse knockout of endogenous genes with simultaneous labeling of the cells undergoing the knockout event, e.g. in the STARS constructs, mCherry expression can be also viewed as GFP knockout. With STARS, the level of sparseness might not be influenced by the locus of gene to be knocked out in the genome.

A major advantage of STARS is to be able to regulate the level of sparseness through controlling the stochastic level of gene activation. How sparse the STARS strategy can reach remains to be tested. It is possible that by combining two mechanisms adjusting reaction kinetics, i.e. by varying the length of lox-flanked DNA fragment and mutagenizing *lox* sites, lower sparseness level can be achieved. Based on the results from the present study, the STARS strategy can be potentially applied to create genetic mosaics for studying the population dosage of genes underlying sporadic genetic diseases, to induce loss of heterozygosity for modeling cancer development and screening for tumor suppressor genes, to trace neuronal circuit and cell fate, as well as to determine cell-autonomous function of a gene, etc. Taken together, as a purely genetic method, STARS can be conveniently and broadly applied, with tissue- or cell-type specificity provided by specific Cre lines.

## Methods

### Generation of STARS constructs and CAGGS-Cre plasmid

All constructs were made using standard molecular cloning methods. Briefly, CMV β-actin enhancer (CAGGS) promoter [19], [20] was digested from pCAGGS-ES plasmid (gift of Dr. Le Ma) using restriction enzyme SpeI plus EcoRI and filled to blunt end at EcoRI site with T4 DNA polymerase (Promega). This fragment was subcloned into pBlue-script SK+/− digested with SpeI plus SmaI. To obtain *lox*-flanked XFP, *loxN* pair was introduced in the primers amplifying mKusabira orange [21] (5′ primer: ctgcagataacttcgtatagtatacctta tacgaagttataccatggtgagtgtgattaa, 3′primer: ctgcagataacttcgtataaggtatactatacgaagttatattaa aaaacctcccacacct) and *lox2272-loxP* pair was introduced in the primers amplifying both membrane bound (M)-GFP(or lynGFP) [22] (5′ primer: agatatcctgcagataacttcgtataggatac tttatacgaagttataccatgggatgtattaaatca, 3′primer: ccatgggaagcttataacttcgtataatgtatgctatac gaagttatgaattcggccggccggcgcgccgaattcgtcgaggccgcgaattaaaaaacc) and mCherry [23] (5′ primer: ccatgggaagcttataacttcgtataggatactttatacgaagttatcacgtgccaccatggtgagcaagggc gagga, 3′ primer: ggaattcctcgagataacttcgtataatgtatgctatacgaagttatagatctgtcgaggccgcg aattaaaaaacc). Sequences underlined are corresponding *lox* sites or their reverse complements. Polymerase chain reaction (PCR) was then performed to generate PstI-*lox2272*-M-GFP-SV40polyA-EcoRI-AscI-FseI-EcoRI-*loxP*-HindIII, PstI-*loxN*-mKO-SV40polyA- *loxN*-PstI and HindIII-*lox2272*-mCherry-SV40polyA-*loxP*-XhoI fragments, which were sequentially inserted downstream of CAGGS promoter in pBlue-script SK+/− by PstI plus HindIII, PstI and HindIII plus XhoI respectively to make STARS 1-1. We then generated multiples of tetra-poly(A) fragment [24] (4 kb, 8 kb, 12 kb) flanked by AscI and FseI in pCR-script plasmid and inserted these fragments or a single poly(A)(1 kb) fragment flanked by EcoRI into the spacer of STARS1-1 by either AscI plus FseI or EcoRI respectively to generate other STARS constructs. To make stable HEK293 cell lines, the entire STARS cassettes was subcloned using XhoI and NotI sites into pcDNA5/FRT expression vector (Invitrogen) previously modified to remove its CMV promoter. CAGGS-Cre construct was made by subcloning Cre cDNA plus HSV-TKpolyA from pACN plasmid [25] to pCAGGS-ES vector using EcoRI plus NotI sites.

### Generation of stable HEK293 cell lines with single-copy STARS integration

HEK293 cell lines stably transfected with single copy of various STARS constructs were made using Flp-In™ system (Invitrogen). Briefly, HEK293 Flp-In cells were cultured in DMEM, containing 10% FCS, 1% penicillin/streptomycin and 100 ug/ml zeocin in a temperature- and humidity-controlled incubator (95% air, 5% CO_2_ as the gas phase) at 37°C. Subconfluent cells were cotransfected with pOG44 (Invitrogen): pcDNA/FRT/STARS at 9∶1 ratio using electroporation methods. 48 hrs after transfection, cells were split into 10 cm tissue culture dishes and selected with 100 ug/ml hygromycin until colonies are visible. Individual colonies were further screened for mKO expression and picked to expand until subconfluence for transfection. Due to the fact that Flp-In system utilized Flp recombinase mediated transgene integration into single Frt site previously inserted in the genome of Flp-In™HEK293 cells, the clones generated should be isogenic and have single copy integration of STARS transgenes. This is further supported by the fact that in all tested cell lines M-GFP-positive and mCherry-positive cells are separate populations after Cre action.

### Cell Transfection, FACS sorting and data analysis

HEK293 Flp-In cells with single copy STARS integration were cultured under conditions described above until subconfluence, and then transfected with CAGGS-Cre plasmid using lipofectamine 2000 (Invitrogen) according to the product manual. Five days after transfection, cells were imaged with a custom-built epi-fluorescence microscope equipped with a 10× objective (Olympus). For each line with desired STARS transgene, around 800 cells were counted for each transfection and data from five independent transfections were averaged to derive the ratio of the number of M-GFP positive versus mCherry positive cells. In several other experiments, we included 1 µM cytosine arabinoside in the medium during transfection to slow down cell proliferation as to limit potential loss of Cre plasmid during cell division. No significant difference was observed between results with and without cytosine arabinoside treatment. In addition, we infected cells with lentivirus expressing CreER under CMV promoter with a MOI (multiplicity of infection) of 8 and cultured cells in the presence of 1.25 µM 4-hydroxy-tamoxinfen. Again, no significant difference was observed compared to the data from Cre plasmid transfection. The following filter sets (Chroma Technology) were used to separate signals between GFP/mKO/mCerry channels without detectable cross-talk: for GFP, excitation 465/37, emission 512/35, and 490DCLP; for mKO: 540/10, 570/20, and 550DCLP; for mCherry, 580/10, 630/50, and 600DCLP. Images were analyzed and edited using Image J software.

Cells were trypsinized after imaging, suspended in PBS and subjected to FACS sorting for M-GFP positive cells. The latter were seeded in 96-well plates at single cell/well for expansion and subsequent transfection. The FACS was performed using a BD FACSAria cell sorter.
